# Kallistatin deficiency exacerbates neuronal damage after cardiac arrest

**DOI:** 10.1038/s41598-024-54415-z

**Published:** 2024-02-21

**Authors:** Hayoung Kim, Gil Joon Suh, Woon Yong Kwon, Kyung Su Kim, Yoon Sun Jung, Taegyun Kim, Heesu Park

**Affiliations:** 1 Department of Emergency Medicine, Seoul National University Hospital, Seoul, Republic of Korea; 2 Research Center for Disaster Medicine, Seoul National University Medical Research Center, Seoul, Republic of Korea; 3 Department of Emergency Medicine, Seoul National University College of Medicine, Seoul, Republic of Korea; 4 Department of Critical Care Medicine, Seoul National University Hospital, Seoul, Republic of Korea

**Keywords:** Diseases, Neurology

## Abstract

The purpose of study was to evaluate that kallistatin deficiency causes excessive production of reactive oxygen species and exacerbates neuronal injury after cardiac arrest. For in vitro study, kallistatin knockdown human neuronal cells were given ischemia–reperfusion injury, and the oxidative stress and apoptosis were evaluated. For clinical study, cardiac arrest survivors admitted to the ICU were divided into the good (CPC 1–2) and poor (CPC 3–5) 6-month neurological outcome groups. The serum level of kallistatin, Nox-1, H_2_O_2_ were measured. Nox-1 and H_2_O_2_ levels were increased in the kallistatin knockdown human neuronal cells with ischemia–reperfusion injury (p < 0.001) and caspase-3 was elevated and apoptosis was promoted (SERPINA4 siRNA: p < 0.01). Among a total of 62 cardiac arrest survivors (16 good, 46 poor), serum kallistatin were lower, and Nox-1 were higher in the poor neurological group at all time points after admission to the ICU (*p* = 0.013 at admission; *p* = 0.020 at 24 h; *p* = 0.011 at 72 h). At 72 h, H_2_O_2_ were higher in the poor neurological group (*p* = 0.038). Kallistatin deficiency exacerbates neuronal ischemia–reperfusion injury and low serum kallistatin levels were associated with poor neurological outcomes in cardiac arrest survivors.

## Introduction

67% of adults and 55% of children who undergo restoration of spontaneous circulation (ROSC) after cardiac arrest die from post-cardiac arrest syndrome and circulatory failure^1^. The neurological outcomes after cardiac arrest are still poor and many surviving patients suffer severe neuronal damage^2,3^. The main mechanisms of brain damage after cardiac arrest are apoptosis and ischemia–reperfusion injury, in other words, the oxygen supply to the tissues is decreased, reactive oxygen species are produced in neuronal cells through the damage-related processes that occur following the reperfusion after ischemia^4,5^. According to previous studies, when reactive oxygen species are excessively produced by NADPH (nicotinamide adenine dinucleotide phosphate) oxidase at the beginning of ischemia–reperfusion injury^6,7^, reactive oxygen-dependent intracellular signalling pathways are activated, which lead to pro-inflammatory cytokines release^8–10^.

The human body has its own antioxidant defense system, that functions to prevent oxidative damage, and the cell damage caused by reactive oxygen species^11,12^. However, when the reactive oxygen species levels exceeds the body's own antioxidant defense capabilities, oxidative stress occurs^13,14^. Compared to the other organs, the brain has a lower concentration of antioxidant enzymes such as superoxide dismutase, catalase, and glutathione peroxidase, and has been found to be very susceptible to oxidative damage^15^. Cardiac arrest occurs Oxygen delivery is dramatically reduced and leads to severe tissue hypoxia. If ischemia is prolonged, reactive oxygen species accumulates in the tissue and reperfusion occurs through cardiopulmonary resuscitation (CPR) and ROSC. In particular, such ischemia–reperfusion injury of neuronal cells causes hypoxic brain damage, and many patients suffer from severe neurological sequelae such as vegetative state or brain death^2^. However, to date, targeted temperature management has been the only method used for treating brain damage by inhibiting the reactive oxygen species production, there are no therapeutic drugs yet^16^. Among the many biochemical markers discovered over recent decades, neuron specific enolase and S-100 protein only indicate the degree of neurological damage^17,18^, but no biomarkers have been developed for use in treatment.

Recent proteomics studies have shown that low serum levels of kallistatin in out-of-hospital cardiac arrest patients are associated with poor neurological outcomes of cardiac arrest survivors^19^. Kallistatin is a kallikrein-binding protein discovered by Chao et al. in 1986. Kallistatin is encoded by the SERPINA4 gene and is found in human plasma^20,21^. Kallistatin is mainly expressed in the liver, but is widely distributed in tissues related to the cardiovascular system, such as the heart, kidneys, and blood vessels^22–24^. Kallistatin is an endogenous serine proteinase inhibitor and has antioxidant functions, it has been found that kallistatin inhibits the production of reactive oxygen species by reducing the activity of NADPH oxidase^25^. Kallistatin also exerts antioxidant effects by promoting the synthesis of endothelial nitric oxide synthase (eNOS), Sirtuin 1 (SIRT1), and forkhead box protein O1 (FoxO1)^25–28^. Kallistatin reduces myocardial ischemia–reperfusion injury by preventing apoptosis and inflammation^29^. A study on these antioxidant and anti-inflammatory effects of kallistatin, reported that lower serum levels of kallistatin are associated with mortality in patients with septic shock^30^ and that treatment with kallistatin reduces organ damage in animal models of sepsis^31^. However, it has not yet been studied how low kallistatin expression aggravates brain damage in ischemia–reperfusion injuries such as cardiac arrest.

So, the purpose of this study was to show that low expression of kallistatin causes excessive production of reactive oxygen species and exacerbates oxidative damage in human neuronal cells with ischemia–reperfusion injury and cardiac arrest survivors with poor neurological outcome.

## Methods

### In vitro study

#### Cell line and kallistatin knockdown human neuronal cells

The human cortical neurons (HCN-2, ATCC^®^ CRL-10742TM, *Homo sapiens* brain encephalitis) used in the experiment. To produce kallistatin knockdown neuronal cells, some of the cultured human neuronal cells (HCN-2) were transfected with small interfering RNA (SERPINA4 siRNA) that inhibited the expression of kallistatin to establish an experimental group. To confirm the transfection efficiency, the mRNA expression of SERPINA4 was confirmed by real time PCR. (Supplement 1A–C).

To establish an ischemia–reperfusion injury model by oxygen–glucose deprivation (OGD) and reoxygenation (Reoxy), human neuronal cells were cultured for 48 h. Then, Dulbecco's modified Eagle medium (DMEM), without glucose (11966025; Thermo Fisher Scientific, Waltham, MA), which is a glucose-deficient medium, was added to the culture plates, and the plates were incubated in a hypoxic chamber consisting of 95% nitrogen (INCO108, Memmert, Schwabach, Germany) for 60 min. After oxygen–glucose deprivation, the culture media was replaced with growth media, and reoxygenated for 23 h in a chamber containing 95% air and 5% carbon dioxide (OGD/Reoxy). The OGD/Reoxy model mimics cerebral ischemia–reperfusion injury and is known to cause brain damage more rapidly than blocking the oxygen supply alone^32^. The cell viability was measured using the tetrazole assay method (MTT assay) with/without OGD/Reoxy processes in control and kallistatin knockdown human neuronal cells. (Supplement. 2A) The cell viability between the OGD/Reoxy group and control group was analyzed according to different oxygen–glucose deprivation times. (Supplement. 2B) Among the various oxygen–glucose deprivation times, the appropriate OGD/Reoxy time was determined to be 60 min, considering that the cell viability measurement was not too low, showing a clear difference from the control group.

#### Kallistatin concentration measurement

The cells were divided into 4 groups: the control siRNA group, the kallistatin knockdown group transfected with SERPINA4 siRNA, the control siRNA group treated with OGD/Reoxy, and the kallistatin knockdown group treated with OGD/Reoxy. Although the number of cells per group was generally small, the concentration of kallistatin was quantitatively measured using the SERPINA4 (Human) ELISA Kit (KA3892; Abnova, Walnut, CA).

#### Measurement of oxidative stress and apoptosis

To measure the intracellular oxidative stress in the four groups, the expression of NADPH oxidase (Nox-1) was measured by western blotting using anti-Nox-1 antibody (Abcam, Catalog number: ab55831). The H_2_O_2_ concentration was measured using a hydrogen peroxide colorimetric detection kit (ADI-907-015, Enzo Life Science, Farmingdale, NY), and apoptosis was measured by western blot using anti-caspase 3 (1:1000; 9664; Cell Signaling, Danvers, MA).

### Clinical study

#### Study settings and designs

For clinical investigation, retrospective observational study was conducted based on prospectively collected data from cardiac arrest survivors and plasma samples. Patients whose spontaneous circulation recovered after cardiac arrest included in the study were admitted to the Emergency Intensive Care Unit (EICU) of a tertiary hospital. The study protocol was approved by the Institutional Review Board of Seoul National University College of Medical/Seoul National University Hospital (IRB number: 2104-179-1214 for the present study). The repository of clinical data and blood samples from patient with cardiac arrest, the use of stored samples were approved by the Institutional Review Board of Seoul National University College of Medical/Seoul National University Hospital(IRB number: 1408-012-599 for the prospective data collection; IRB number: 1707-012-865 for the blood sample collection). Written informed consent was obtained from each patient or legally authorized representative. The repository protocol for patients with cardiac arrest is registered at ClinicalTrials.gov (NCT01670383). For research involving human participants, all authors identified the committee that approved the research, confirm that all research was performed in accordance with SNUH HRPP SOP Ver. 4.0 guideline (http://hrpp.snuh.org). Research involving human research participants was performed in accordance with the Declaration of Helsinki.

Data were collected from patients with cardiac arrest who had been admitted to the emergency intensive care unit (EICU) of a tertiary referral hospital (Seoul national university hospital) from January 1, 2016 to February 28, 2021. Inclusion Criteria was a patient over the age of 18 who recovered spontaneous circulation after cardiac arrest in the emergency room. Among the resuscitated patients after cardiac arrest, patients with written consent to provide clinical information and collect samples were included in the study. Exclusion criteria were as follows: patients under the age of 18, no written informed consent, incompletion of blood sampling, insufficient blood samples for analysis, and neurological outcome was not followed for 6 months. Blood samples were collected from patients with cardiac arrest three times after admission according to a standardized treatment protocol, i.e. at admission, 24 h after admission, and 72 h after admission. Blood samples were stored at − 80 °C until the analysis.

#### Clinical management and data collection

Patients were provided with hemodynamic support and appropriate management according to the international guidelines for management of post cardiac arrest. According to the 6-month cerebral performance category (CPC) scale^33^, the patients were divided into good (CPC 1–2) and poor (CPC 3–5) neurological outcome groups. At the time of admission to the ICU, we collected demographic data and laboratory test results, and Glasgow coma scale. We also collected laboratory test results and Glasgow coma scale at 24 h and 72 h after admission.

#### Measurement of kallistatin level, oxidative stress and apoptosis

Serum levels of kallistatin were measured by enzyme-linked immunosorbent assay (ELISA) in duplicate for the blood samples collected from the patients at admission, 24 h, and 72 h. To Measure of kallistatin level, oxidative stress and apoptosis, human SERPINA4/Kallistatin DuoSet ELISA (R&D Systems, catalog number DY1669, DY008, Minneapolis, MN), human NOX-1 ELISA kit (NOVUS biologicals™, Catalog number: NBP2-76746), hydrogen peroxide colorimetric detection kit (Abcam, Catalog number: Ab102500) were used.

#### Statistical analysis

The statistical analysis of the experimental results was performed using ANOVA with the Tukey post-hoc test method. Clinical data were presented as mean ± standard deviation, median (interquartile range), or n (%). Categorical data were compared using chi-square tests or Fisher's exact tests, and continuous data were compared using Student's t-tests or Mann–Whitney U tests as appropriate. *P* values of < 0.05 were considered to be statistically significant, and the significance levels quoted are two sided. The statistical analyses were conducted using SPSS version 21.0 for Windows (SPSS, Chicago, IL).

## Results

### In vitro study

#### Measurement of cell viability with/without OGD-Reoxy

**Figure 1:**
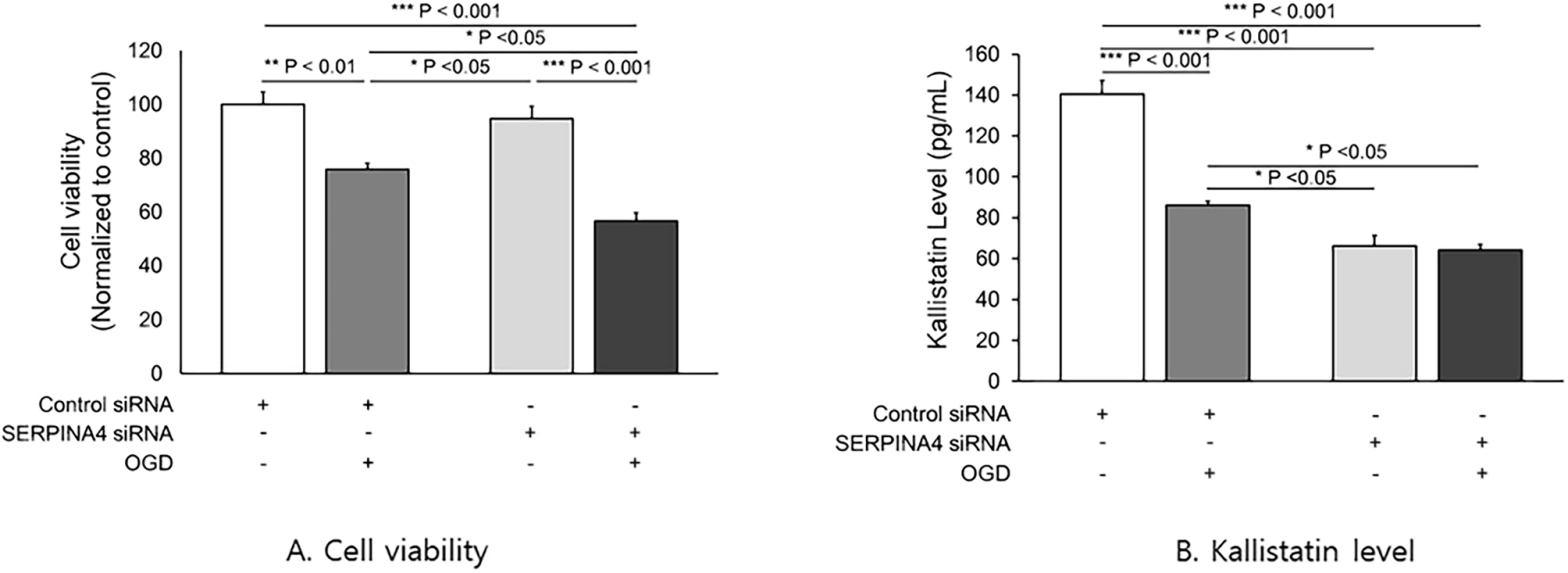
Cell viability and kallistatin level of OGD/Reoxy-treated control and kallistatin knockdown HCN-2 cells. (**A**) The cell viability was measured with/without OGD/Reoxy processes in control and kallistatin knockdown human neuronal (HCN-2) cells. In both the control and kallistatin knockdown cells, exposure to OGD/Reoxy decreased cell viability. (Control siRNA: p < 0.01, SERPINA4 siRNA: p < 0.001). (**B**) The expression of kallistatin was decreased the most in the kallistatin knockdown HCN-2 cells with OGD/Reoxy. OGD: oxygen–glucose deprivation, reoxy: reoxygenation.

In both the control siRNA and SERPINA4 siRNA-transfected groups, exposure to OGD/Reoxy decreased cell viability. (Control siRNA: *p* < 0.01, SERPINA4 siRNA: *p* < 0.001) (Fig. 1A). Among the groups, the decrease in the cell viability after exposure to OGD/Reoxy was more pronounced when the OGD/Reoxy was conducted in kallistatin knockdown neuronal cells.

#### Measurement of kallistatin concentrations

The expression of kallistatin was decreased in the control siRNA-transfected cell group with OGD/Reoxy compared to the control siRNA-transfected cells without OGD/Reoxy (*p* < 0.001). In addition, compared to the control siRNA group, the SERPINA4 siRNA group showed that kallistatin expression was suppressed (*p* < 0.001). This expression level was lower than the level of kallistatin produced when the control siRNA group was subjected to OGD/Reoxy (*p* < 0.05). However, kallistatin knockdown neuronal cells (SERPINA4 siRNA) did not show a significant difference in the concentration of kallistatin after OGD/Reoxy (Fig. 1B).

#### Measurement of oxidative stress and apoptosis

**Figure 2:**
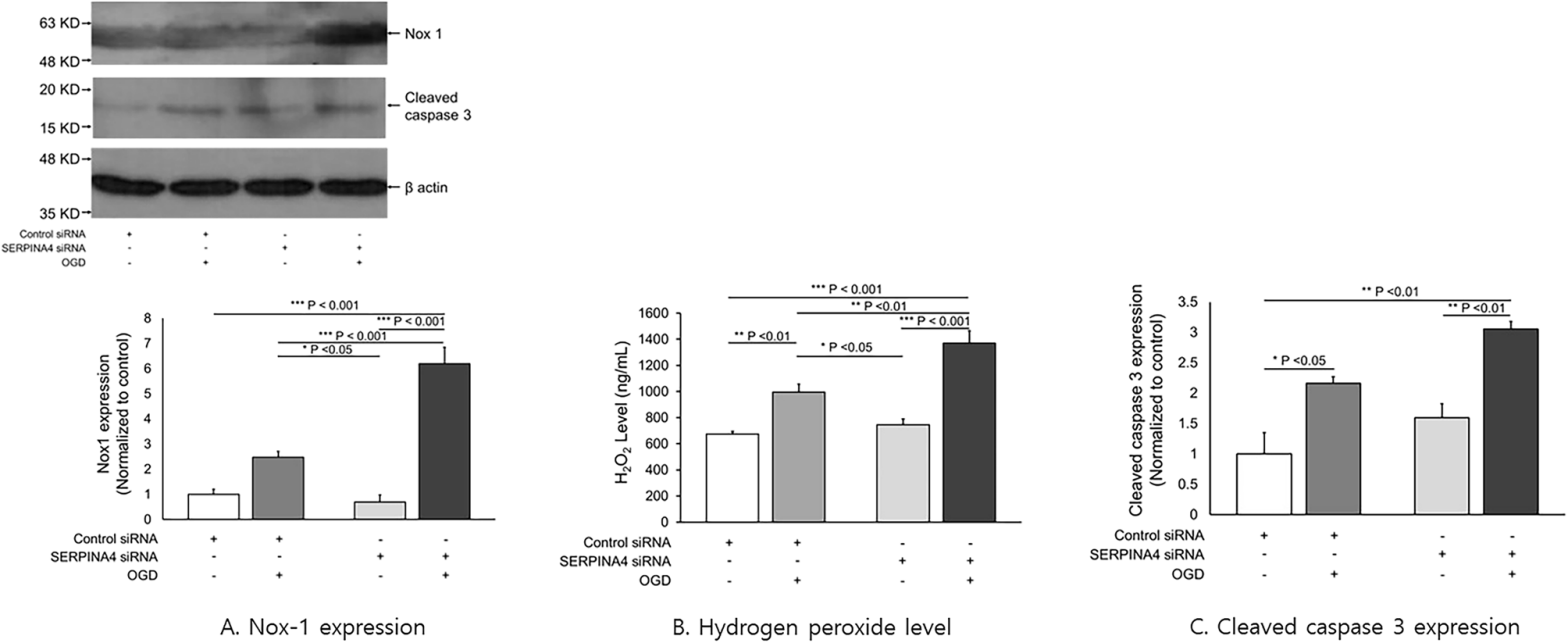
Nox-1, Hydrogen peroxide, Cleaved caspase 3 in control and kallistatin knockdown HCN-2 cells after exposure to OGD/Reoxy. Nox-1 expression and H_2_O_2_ levels were increased in the kallistatin knockdown human neuronal cells with OGD/Reoxy (p < 0.001) and cleaved caspase-3 expression was elevated and apoptosis was promoted (SERPINA4 siRNA: p < 0.01). OGD: oxygen–glucose deprivation, Reoxy: reoxygenation. Original blots/gels are presented in Supplementary Fig. 3.

The Nox-1 expression was increased in the kallistatin knockdown cells with OGD/Reoxy compared to kallistatin knockdown cells without OGD/Reoxy *(p* < 0.001). In addition, Nox-1 expression was further increased in the kallistatin knockdown cells with OGD/Reoxy compared with the control cells with OGD/Reoxy (*p* < 0.001) (Fig. 2A). The concentration of hydrogen peroxide was higher in the control siRNA group with OGD/Reoxy than in the control siRNA group without OGD/Reoxy (*p* < 0.01). The concentration of hydrogen peroxide was also increased after the kallistatin knockdown cells were exposed to OGD/Reoxy (*p* < 0.001) (Fig. 2B). Cleaved caspase 3 expression was increased in both the control siRNA and SERPINA4 siRNA with OGD/Reoxy. (Control siRNA: *p* < 0.05, SERPINA4 siRNA: *p* < 0.01) (Fig. 2C).

### Clinical investigation

**Figure 3:**
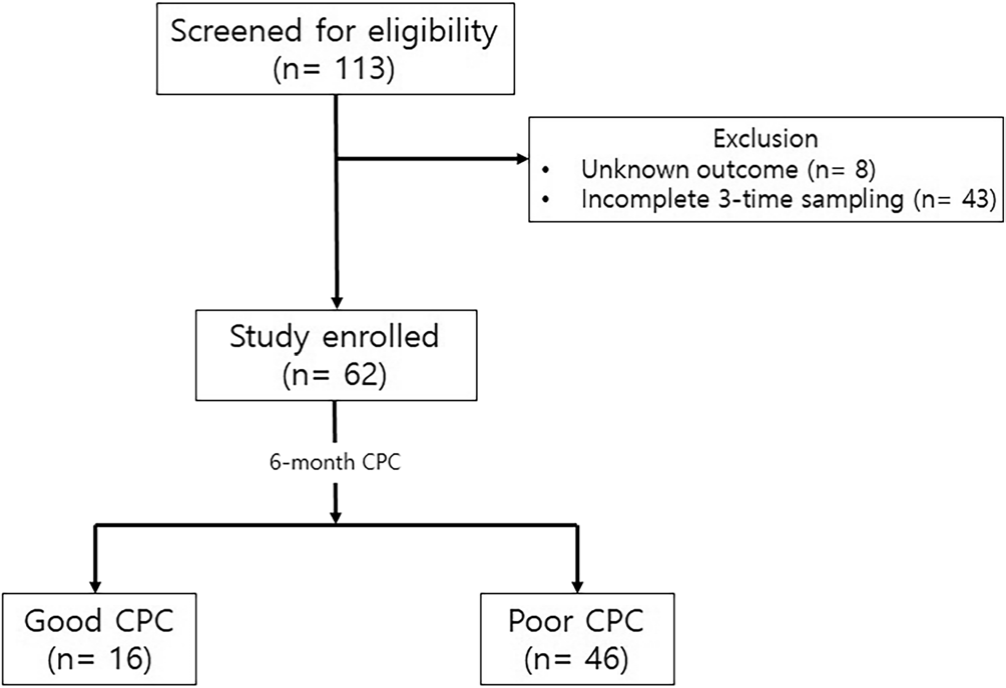
Clinical investigation flow diagram. Out of 113 OHCA patients, a total of 62 patients were selected as the final study subjects. 16 patients had a good neurological outcome (CPC 1–2) and 46 patients had a poor neurological outcome (CPC 3–5) 6 months after cardiac arrest. OHCA: out of hospital cardiac arrest, CPC: cerebral performance category.

One hundred thirteen patients were screened for eligibility during the study period. Fifty-one patients were excluded according to pre-specified exclusion criteria, and 62 patients were enrolled for the study after all exclusions (Fig. 3). Baseline demographics and clinical characteristics for the patients are shown in Tables 1 and 2. There were no differences between the good CPC group and poor CPC group in terms of age, sex, and underlying co-morbidity. The Good CPC group showed more shockable rhythm and causes of cardiac problems (*p* < 0.001). The Poor CPC group had more cardiac arrest due to respiratory cause and longer ICU length of stay. At admission time, 24 h, and 72 h, serum lactate levels were higher in the poor CPC group than in the good CPC group (*p* < 0.05). At 24 and 72 h, serum NSE levels were also higher in the poor CPC group than in the good CPC group (*p* < 0.001).

**Table 1:** Baseline demographics.

	GOOD CPC (CPC 1–2) (N = 16)	POOR CPC (CPC 3–5) (N = 46)	*P* value
Age, years	68.2 ± 12.8	69.1 ± 18.5	0.430
Male sex, n(%)	10 (87.5%)	29 (63.0%)	0.114
Baseline CPC			0.052
1–2	16 (100%)	36 (78.3%)	
3	0	12 (26.1%)	
Underlying disease			
HTN	11 (68.7%)	20 (43.5%)	0.082
DM	6 (37.5%)	14 (30.4%)	0.757
Chronic liver ds.	1 (6.3%)	2 (4.3%)	0.599
Chronic heart ds.	7 (43.7%)	11 (23.9%)	0.119
Chronic lung ds.	1 (6.3%)	7 (15.2%)	0.330
Chronic kidney ds.	2 (12.5%)	8 (17.4%)	0.014
Chronic neurologic ds.	1 (6.3%)	14 (30.4%)	0.047
Solid malignancy			0.154
Yes, without meta	1 (6.3%)	1 (2.1%)	
Yes, with meta	0 (0%)	8 (17.4%)	
NED	3 (18.7%)	4 (8.7%)	
Initial rhythm			0.001
VF	10 (87.5%)	1 (2.1%)	0.001
Pulseless VT	2 (12.5%)	6 (13.0%)	0.272
PEA	4 (25.0%)	20 (43.5%)	0.242
Asystole	0 (0%)	17 (37%)	0.003
CAG/PCI			0.001
No	6 (37.5%)	40 (21.7%)	0.001
CAG, not PCI	4 (25.0%)	2 (4.3%)	0.034
CAG with PCI	6 (37.5%)	4 (8.7%)	0.014
Total no flow time	0.5 ± 1.37	4.35 ± 8.22	0.041
Total low flow time	11.31 ± 5.53	23.74 ± 13.25	0.001
Arrest etiology			0.001
Medical-(cardiac)	12 (75.0%)	9 (19.7%)	0.001
Medical-(respiratory)	1 (6.3%)	25 (54.3%)	0.001
Medical-(other etiologies)	3 (18.7%)	8 (17.4%)	1.000
Non-medical	0 (0%)	4 (8.7%)	0.565

Data are presented as mean ± SD, median (IQR), or n (%).

NED: no evidence of disease.

**Table 2:** Baseline demographics after ICU admission.

	GOOD CPC (CPC 1–2) (N = 16)	POOR CPC (CPC 3–5) (N = 46)	*P* value
ICU LOS	8.75 ± 5.10	18.11 ± 13.24	0.004
ICU discharge survival	16 (100%)	22 (47.8%)	0.001
28D mortality (death)	0 (0%)	19 (41.3%)	0.001
Parameters at admission			
GCS	5.81 ± 3.39	3.66 ± 1.74	0.001
Blood Hb, g/dL	11.75 (10.20–14.05)	11.6 (8.60–13.05)	0.584
Blood WBC count, 1000/μL	12.16 (9.19–14.71)	13.63 (11.05–20.11)	0.152
Serum creatinine, mg/dL	1.20 (0.96–1.47)	1.16 (0.89–1.76)	0.705
Serum hs-CRP, mg/dL	0.36 (0.17–3.09)	0.82 (0.18–7.21)	0.579
Serum troponin I, ng/mL	0.59 (0.12–1.62)	0.22 (0.07–1.02)	0.166
Serum lactate, mmol/L	3.25 (1.30–4.78)	5.55 (2.85–8.65)	0.007
Serum NSE, ng/mL	36.05 (23.67–57.73)	46.03 (31.00–69.88)	0.228
Parameters at 24 h			
GCS	5.18 ± 3.81	3.58 ± 1.87	0.043
Blood Hb, g/dL	11.35 (9.60–13.30)	10.10 (8.70–13.36)	0.381
Blood WBC count, 1000/μL	9.86 (7.43–13.25)	12.36 (9.94–18.31)	0.028
Serum creatinine, mg/dL	1.04 (0.59–1.33)	1.06 (0.57–2.20)	0.797
Serum hs-CRP, mg/dL	4.14 (2.90–11.96)	11.02 (5.82–15.32)	0.012
Serum troponin I, ng/mL	0.60 (0.34–2.82)	0.37 (0.11–1.89)	0.241
Serum lactate, mmol/L	1.30 (0.75–3.05)	2.60 (1.48–4.53)	0.024
Serum NSE, ng/mL	32.79 (21.02–43.94)	106.55 (40.82–358.90)	0.001
Parameters at 72 h			
GCS	10.93 ± 3.68	4.91 ± 2.78	0.001
Blood Hb, g/dL	10.30 (8.90–12.05)	9.75 (8.70–11.43)	0.535
Blood WBC count, 1000/μL	9.07 (6.87–11.74)	11.43 (8.76–15.84)	0.082
Serum creatinine, mg/dL	0.99 (0.62–1.36)	1.10 (0.63–2.50)	0.834
Serum hs-CRP, mg/dL	6.82 (4.44–11.65)	11.19 (7.33–22.88)	0.010
Serum troponin I, ng/mL	0.26 (0.08–1.30)	0.24 (0.07–1.00)	0.872
Serum lactate, mmol/L	1.20 (0.95–1.55)	1.65 (1.28–2.58)	0.017
Serum NSE, ng/mL	20.47 (13.14–33.28)	219.40 (66.80–370.00)	0.001

Data are presented as mean ± SD, median (IQR), or n (%).

WBC, white blood cell; IQR, interquartile range.

#### Measurement of serum kallistatin, oxidative stress and apoptosis

**Figure 4:**
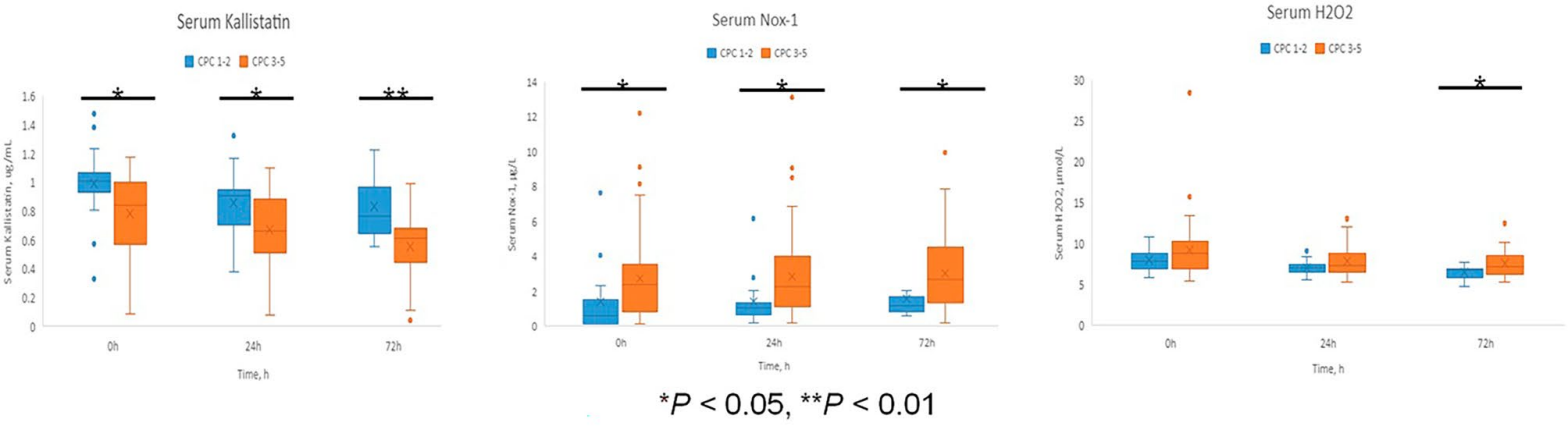
Serum kallistatin, Nox-1, hydrogen peroxide level among 6-month good CPC (CPC 1–2) and poor CPC (CPC 3–5). Serum levels of kallistatin, NADPH oxidase, and H_2_O_2_ were measured both good (blue line) and poor CPC (orange line) group at admission time, 24 h, and 72 h. Serum kallistatin levels were lower in poor CPC groups and Nox-1 levels were higher at all time points (0.60 μg/L vs. 2.41 μg/L, *p* = 0.013 at admission; 1.08 μg/L vs. 2.27 μg/L, *p* = 0.020 at 24 h; 1.17 μg/L vs. 2.67 μg/L, *p* = 0.011 at 72 h). At 72 h, H_2_O_2_ levels were higher in poor CPC groups (6.82 μmol/L vs. 7.27 μmol/L, *p* = 0.038). CPC: cerebral performance category.

Serum levels of kallistatin, NADPH oxidase, and H_2_O_2_ were measured both good and poor CPC group at admission time, 24 h, and 72 h (Table 3). Compared with the good CPC group, serum kallistatin levels were lower in the poor CPC group at all time points (1.01 μg/mL vs. 0.84 μg/mL, *p* = 0.011 at admission; 0.91 μg/mL vs. 0.67 μg/mL, *p* = 0.034 at 24 h; 0.77 μg/mL vs. 0.62 μg/mL, *p* = 0.001 at 72 h), and serum Nox-1 levels were higher in the poor CPC group at all time points (0.60 μg/L vs. 2.41 μg/L, *p* = 0.013 at admission; 1.08 μg/L vs. 2.27 μg/L, *p* = 0.020 at 24 h; 1.17 μg/L vs. 2.67 μg/L, *p* = 0.011 at 72 h). Compared to the good CPC group, H_2_O_2_ level showed no difference at admission and 24 h (7.91 μmol/L vs. 8.83 μmol/L, *p* = 0.21 at admission; 7.05 μmol/L vs. 7.29 μmol/L, *p* = 0.247 at 24 h ), but was higher at 72 h (6.82 μmol/L vs. 7.27 μmol/L, *p* = 0.038) (Fig. 4).

**Table 3:** Comparison of serum levels of Kallistatin, Nox-1, H_2_O_2_ among 6-month good CPC (CPC 1–2) and poor CPC (CPC 3–5).

	GOOD CPC (CPC 1–2) (N = 16)	POOR CPC (CPC 3–5) (N = 46)	*P* value^†^
At admission			
Kallistatin, μg/mL	1.01 (0.93–1.11)	0.84 (0.57–1.00)	0.011
Nox-1, μg/L	0.60 (0.16–2.02)	2.41 (0.76–3.61)	0.013
H_2_O_2_, μmol/L	7.91 (6.63–9.10)	8.83 (6.90–10.30)	0.210
At 24 h			
Kallistatin, μg/mL	0.91 (0.70–0.98)	0.67 (0.49–0.89)	0.034
Nox-1, μg/L	1.08 (0.62–1.43)	2.27 (1.08–4.09)	0.020
H_2_O_2_, μmol/L	7.05 (6.45–7.64)	7.29 (6.51–8.90)	0.247
At 72 h			
Kallistatin, μg/mL	0.77 (0.64–1.02)	0.62 (0.43–0.70)	0.001
Nox-1, μg/L	1.17 (0.82–1.78)	2.67 (1.30–4.62)	0.011
H_2_O_2_, μmol/L	6.82 (5.83–7.07)	7.27 (6.23–8.69)	0.038

Data are presented as median (IQR).

Nox-1, nicotinamide adenine dinucleotide phosphate oxidase-1; IQR, interquartile range.

^†^P values for Mann–Whitney U tests between good CPC survivors and poor CPC survivors.

## Discussion

The purpose of this study was to investigate the role of kallistatin in ischemia–reperfusion induced neuronal injury, and this study particularly focused on the antioxidant effect of kallistatin on neuronal damage. In the in-vitro study mimicking cardiac arrest, kallistatin knockdown cells exposed to OGD/Reoxy showed an increase in Nox-1 expression, H_2_O_2_ levels, and caspase-3 expression. In the clinical study, the serum kallistatin levels were lower and Nox-1 levels were higher in poor CPC neurological outcome group at admission, 24 h and 72 h. At 72 h, H_2_O_2_ levels were higher in the poor CPC group compared with the good CPC group. In the basic demography and clinical characteristics of cardiac arrest patients, the patient's initial shockable rhythm, cause of cardiac arrest, lactic acid levels, and NSE levels differed between the good and poor CPC groups. Among these clinical variables, only kallistatin concentration was emphasized in this study because a previous our proteomics study showed that low serum kallistatin levels in cardiac arrest patients were associated with poor neurological outcomes in cardiac arrest survivors. In this study, we investigated the mechanism by which kallistatin deficiency worsens neuronal ischemic damage in an in vitro study and focused on examining the effect of kallistatin differences among these clinical variables on neurological prognosis in post cardiac arrest patients. In order to develop a treatment, it must be proven that the lack of the substance is significantly related to a neurological poor prognosis or that supplementation of the substance improves the neurological outcomes^34,35^. Knowing why kallistatin deficiency worsens neuronal ischemic injury could lay the foundation for developing kallistatin as a treatment that can protect neuronal cells and reduce brain ischemic damage in the future.

Ischemia–reperfusion injury refers to a series of clinical and experimental results caused by reperfusion after ischemia caused by a decrease in oxygen supply to tissues^5^. Ischemia–reperfusion injury is known to be involved in the pathophysiology of post-cardiac arrest syndrome, reperfusion after myocardial infarction and stroke, sepsis, severe trauma, and postoperative complications^36^. Various mechanisms that induce ischemia–reperfusion injury have been suggested through previous studies, and it is known that oxidative stress caused by increased production of reactive oxygen species plays an important role^4,11^. In this study, ischemia–reperfusion injury was induced in human neuronal cells through 60 min of oxygen–glucose deprivation and 23 h of reoxygenation to create a mimic situation with neuronal injury in patients with ROSC after cardiac arrest in in-vitro experiments. In the previous study, OGD/Reoxy of kallistatin knockdown cells using human umbilical vein endothelial cells for ischemia–reperfusion injury, 90 min of oxygen–glucose deprivation followed by 22.5 h of reoxygenation treatment^37^. In this experiment, compared to the control group, the cell viability gradually decreased as the oxygen glucose deprivation time increased for 30 min, 60 min, and 90 min of OGD/Reoxy. The OGD/Reoxy time was set to 60 min showing a clear difference from the control group and considering that the measuring cell viability value was not too low.

Kallistatin is mainly expressed in the liver, and widely distributed in tissues relevant to cardiovascular function, including the heart, kidney and blood vessels^21–24^. Different from human endothelial cells^37^, it took a long time to cultivate human neuronal cells, and the number of the cells was not large. The level of kallistatin measured in kallistatin knockdown neuronal cells was 65.9 ρg/ml, and when the kallistatin knockdown neuronal cells with OGD/Reoxy was 64.0 ρg/ml, which was very low, making it difficult to identify a significant difference between the two groups. Since the amount of kallistatin protein expressed by the SERPINA4 gene is very low in the brain compared to the liver and kidney, previous studies have mainly focused on in-vitro and animal model studies on endothelial cells, cardiac remodeling, hypertension, and renal disease^38–41^. This is the first study confirm actual kallistatin levels, oxidative stress, and apoptosis in human neuronal cells with ischemia–reperfusion injury and cardiac arrest survivors.

Through this study, it was found that when ischemia–reperfusion injury occurs in human neuronal cells with kallistatin deficiency, the expression of Nox-1 that makes reactive oxygen species, increased and the levels of H_2_O_2_ and cleaved caspase 3 expression also increased. Also, the low concentration of serum kallistatin was related to poor neurological outcomes in out-of-hospital cardiac arrest survivors. As mentioned earlier, in order to become a therapeutic substance, it is necessary to prove that a lack of the substance can worsen the neurological outcome. In this study, kallistatin knockdown cell experiments and clinical data found that kallistatin deficiency was associated with neuronal damage and poor neurological outcome. Therefore, clinical and in-vitro experiment results indicate that in the patients with cardiac arrest, kallistatin can be a potential prognostic indicator and therapeutic agent for the patient's neurological prognosis.

This study has several limitations. First, compared to previous studies, in this study, only NADPH oxidase and H_2_O_2_ levels, caspase-3 expression were measured for oxidative stress and apoptosis, and proteins related to various pathways could not be identified. Previous studies have been reported that the kallistatin plays an important role in stimulating the expression of the antioxidant enzymes eNOS, SIRT1 and catalase in endothelial cells^42,43^. Kallistatin also and antagonized TNFα-mediated suppression of eNOS synthesis and NO formation in EPCs^44^. The mechanisms of NO and eNOS are mainly related to antioxidant effects in cardiomyocytes or vascular endothelial cells, and this study confirmed the relationship between oxidative stress by NADPH oxidase and organ, especially neuronal injury. It seems that a more accurate pathway could have been identified if the expression of various oxidative and apoptosis-related proteins was compared. Second, the relatively small sample size limits the interpretation of the present study. This study was a single center study that included cardiac arrest survivors from 2016 to February 2021, but only 62 patients were included. Also, we excluded the patients without blood samples from any of 3 time points because of death or transfer to other departments. There might be risks for selection bias according to the exclusion criteria. Despite the small sample size, the serum kallistatin levels at all time points were significantly lower in the poor CPC group. We expect further multicenter studies with larger scale may consolidate our results. Third, it was not possible to actually find out whether oxidative stress could be reduced when kallistatin was administered as a therapeutic agent. However, it was confirmed that oxidative stress and inflammation decreased when kallistatin was re-administered in animal models of arthritis, high blood pressure, myocardial ischemia, and sepsis^30,44,45^. This study is thought to have increased oxidative stress due to NADPH oxidase activity, but if a positive feedback experiment was conducted after the OGD/Reoxy process, the relationship between kallistatin and neuronal oxidative injury could have been more clearly. However, in this study, the preparation of an adenovirus vector containing human kallistatin and the purification of recombinant kallistatin were too complicated, and it was difficult to determine the timing of kallistatin administration in the OGD/Reoxy model, so the experiment to confirm the effect of kallistatin administration were not conducted. In the future, if kallistatin is administered to cardiac arrest survivors, cardiac arrest animal models, and ischemic reperfusion damage of neuronal cells, the relationship between kallistatin and neuronal oxidation damage can be more clearly identified and used as predictive indicators of treatment and neurological outcome.

## Conclusions

In in vitro study of human neuronal cells underwent OGD/reoxy treatment, we found that kallistatin deficiency contributed to increased cellular ischemia–reperfusion injury. Clinical study of cardiac arrest survivors also showed low serum kallistatin levels were associated with their poor neurological outcomes. Our findings suggest kallistatin may be considered as a biomarker to assess neuronal injury and to predict neurological outcomes in cardiac arrest survivors. Furthermore, future studies determining neuroprotective mechanism of kallistatin may help develop adjunctive therapeutic strategies to improve neurological outcomes after cardiac arrest.

### Supplementary Information

Supplementary Information.

## Data Availability

All data generated or analyzed during this study are available from the corresponding author on reasonable request.

## References

[1] Nadkarni VM (2006). First documented rhythm and clinical outcome from in-hospital cardiac arrest among children and adults. JAMA..

[2] Lim C, Alexander MP, LaFleche G, Schnyer DM (2004). The neurological and cognitive sequelae of cardiac arrest. Neurology..

[3] Wachelder EM (2009). Life after survival: Long-term daily functioning and quality of life after an out-of-hospital cardiac arrest. Resuscitation..

[4] Bartos JA, Debaty G, Matsuura T, Yannopoulos D (2014). Post-conditioning to improve cardiopulmonary resuscitation. Curr. Opin. Crit. Care..

[5] Dorweiler B (2007). Ischemia-reperfusion injury: Pathophysiology and clinical implications. Eur. J. Trauma Emerg. Surg..

[6] Kroemer G, Galluzzi L, Brenner C (2007). Mitochondrial membrane permeabilization in cell death. Physiol. Rev..

[7] Kleikers PW (2012). NADPH oxidases as a source of oxidative stress and molecular target in ischemia/reperfusion injury. J. Mol. Med..

[8] Cuzzocrea S, Riley DP, Caputi AP, Salvemini D (2001). Antioxidant therapy: A new pharmacological approach in shock, inflammation, and ischemia/reperfusion injury. Pharmacol. Rev..

[9] Siesjo BK (1995). Glutamate, calcium, and free radicals as mediators of ischemic brain damage. Ann. Thorac. Surg..

[10] Ferrari RS, Andrade CF (2015). Oxidative stress and lung ischemia-reperfusion injury. Oxid. Med. Cell Longev..

[11] Espinosa-Diez C (2015). Antioxidant responses and cellular adjustments to oxidative stress. Redox Biol..

[12] Mourao MM, Dinguirard N, Franco GR, Yoshino TP (2009). Role of the endogenous antioxidant system in the protection of *Schistosoma **mansoni* primary sporocysts against exogenous oxidative stress. PLoS Negl. Trop. Dis..

[13] Ray PD, Huang BW, Tsuji Y (2012). Reactive oxygen species (ROS) homeostasis and redox regulation in cellular signaling. Cell Signal..

[14] Schieber M, Chandel NS (2014). ROS function in redox signaling and oxidative stress. Curr. Biol..

[15] Adibhatha RM, Hatcher JF (2010). Lipid oxidation and peroxidation in CNS health and disease: From molecular mechanisms to therapeutic opportunities. Antioxid. Redox Signal..

[16] Graves PR, Haystead TA (2002). Molecular biologist's guide to proteomics. Microbiol. Mol. Biol. Rev..

[17] Stammet P (2015). Neuron-specific enolase as a predictor of death or poor neurological outcome after out-of-hospital cardiac arrest and targeted temperature management at 33 degrees C and 36 degrees C. J. Am. Coll. Cardiol..

[18] Shinozaki K (2009). S-100B and neuron-specific enolase as predictors of neurological outcome in patients after cardiac arrest and return of spontaneous circulation: A systematic review. Crit. Care..

[19] Jung YS (2018). Low serum Kallistatin level was associated with poor neurological outcome of out-of-hospital cardiac arrest survivors: Proteomics study. Resuscitation..

[20] Chao J, Tillman DM, Wang MY, Margolius HS, Chao L (1986). Identification of a new tissue-kallikrein-binding protein. Biochem. J..

[21] Zhou GX, Chao L, Chao J (1992). Kallistatin: A novel human tissue kallikrein inhibitor. Purification, characterization, and reactive center sequence. J. Biol. Chem..

[22] Chao J, Chao L (1995). Biochemistry, regulation and potential function of kallistatin. Biol. Chem. Hoppe Seyler..

[23] Chen LM, Song Q, Chao L, Chao J (1995). Cellular localization of tissue kallikrein and kallistatin mRNAs in human kidney. Kidney Int..

[24] Wolf WC, Harley RA, Sluce D, Chao L, Chao J (1999). Localization and expression of tissue kallikrein and kallistatin in human blood vessels. J. Histochem. Cytochem..

[25] Guo Y (2015). Kallistatin inhibits TGF-beta-induced endothelial-mesenchymal transition by differential regulation of microRNA-21 and eNOS expression. Exp. Cell Res..

[26] Shen B, Smith RS, Hsu YT, Chao L, Chao J (2009). Kruppel-like factor 4 is a novel mediator of Kallistatin in inhibiting endothelial inflammation via increased endothelial nitric-oxide synthase expression. J. Biol. Chem..

[27] Shen B (2010). Kallistatin attenuates endothelial apoptosis through inhibition of oxidative stress and activation of Akt-eNOS signaling. Am. J. Physiol. Heart Circ. Physiol..

[28] Shen B, Chao L, Chao J (2010). Pivotal role of JNK-dependent FOXO1 activation in downregulation of kallistatin expression by oxidative stress. Am. J. Physiol. Heart Circ. Physiol..

[29] Chao J (2006). Novel role of kallistatin in protection against myocardial ischemia-reperfusion injury by preventing apoptosis and inflammation. Hum. Gene Ther..

[30] Kim T (2018). Lower serum kallistatin level is associated with 28-day mortality in patients with septic shock. J. Crit. Care..

[31] Li P (2015). Kallistatin treatment attenuates lethality and organ injury in mouse models of established sepsis. Crit. Care..

[32] Goldberg MP, Choi DW (1993). Combined oxygen and glucose deprivation in cortical cell culture: Calcium-dependent and calcium-independent mechanisms of neuronal injury. J. Neurosci..

[33] Jennett B, Bond M (1975). Assessment of outcome after severe brain damage. Lancet..

[34] Hughes JP, Rees S, Kalindjian SB, Philpott KL (2011). Principles of early drug discovery. Br. J. Pharmacol..

[35] Schenone M, Dančik V, Wagner BK, Clemons PA (2013). Target identification and mechanism of action in chemical biology and drug discovery. Nat. Chem. Biol..

[36] Kalogeris T, Baines CP, Krenz M, Korthuis RJ (2012). Cell biology of ischemia/reperfusion injury. Int. Rev. Cell Mol. Biol..

[37] Um, Y. W. & Suh, G. J. Role of Kallistatin in oxygen-glucose deprivation and reoxygenation in human umbilical vein endothelial cell. *PhD Thesis* (Seoul National University, 2019). 10.15441/ceem.23.106 PMC11009709 38204159

[38] Chai KX (1997). Molecular cloning and expression of rat kallistatin gene. Biochim. Biophys. Acta..

[39] Wang DZ, Song Q, Chen LM, Chao L, Chao J (1996). Expression and cellular localization of tissue kallikrein-kinin system in human adrenal gland. Am. J. Physiol..

[40] Chao J, Guo Y, Chao L (2018). Protective role of endogenous kallistatin in vascular injury and senescence by inhibiting oxidative stress and inflammation. Oxid. Med. Cell Longev..

[41] Yao Y (2018). Reduced plasma kallistatin is associated with the severity of coronary artery disease, and kallistatin treatment attenuates atherosclerotic plaque formation in mice. J. Am. Heart Assoc..

[42] Gao L (2014). Novel role of kallistatin in vascular repair by promoting mobility, viability, and function of endothelial progenitor cells. J. Am. Heart Assoc..

[43] Shen B, Hagiwara M, Yao YY, Chao L, Chao J (2008). Salutary effect of kallistatin in salt-induced renal injury, inflammation, and fibrosis via antioxidative stress. Hypertension..

[44] Chao J, Li P, Chao L (2017). Kallistatin: Double-edged role in angiogenesis, apoptosis and oxidative stress. Biol. Chem..

[45] Gao L, Yin H, Smith RS, Chao L, Chao J (2008). Role of kallistatin in prevention of cardiac remodeling after chronic myocardial infarction. Lab. Investig..

