# Supplementary material for: Kallistatin deficiency exacerbates neuronal damage after cardiac arrest
Source: Sci Rep. 2024 Feb 21;14:4279. doi: 10.1038/s41598-024-54415-z (PMC10881987; doi:10.1038/s41598-024-54415-z)
Supplement: Supplementary file 1 — Supplementary Information. [file 41598_2024_54415_MOESM1_ESM.docx]

**Supplement 1.**

**A. Cell line and cell culture**

The human cortical neurons (HCN-2, ATCC® CRL-10742TM, *Homo sapiens* brain encephalitis) used in the experiment were purchased from ATCC (American Type Culture Collection, Manassas, VA) and the cell were cultured in Dulbecco's modified Eagle's medium (DMEM) and supplemented with 4mM L-glutamine, 1.5 g/L sodium bicarbonate, 4.5 g/L glucose, and 10% fetal bovine serum. All the cell cultures were maintained in a humidified environment of 5% V/V CO_2_ in air at 37°C.

**B. Kallistatin knockdown human neuronal cells**

Some of the cultured human neuronal cells (HCN-2) were transfected with small interfering RNA (siRNA) that inhibited the expression of kallistatin to establish an experimental group. HCN-2 neurons were cultured in Accell siRNA delivery medium and incubated with siRNA for 72 hours before being subjected to oxygen-glucose deprivation. Knockdown was performed using Accell Human SERPINA4 siRNA-SMARTpool (50 nM, Dharmacon, Lafayette, CO) and RISC-Free siRNA (E-016371-00-0050; Dharmacon) was used as the control siRNA. To confirm the transfection efficiency, the mRNA expression of SERPINA4 was confirmed by real time PCR.

**C. Knockdown expression of the SERPINA4 gene in human neuronal cells**

The kallistatin knockdown neuronal cell group, which was transfected with SERPINA4 siRNA, showed significantly lower mRNA expression of SERPINA4 compared to the control cell group, which was transfected with Control siRNA, and the neuronal cell group, which was not transfected. This approach produced a kallistatin knockdown human neuron cells.
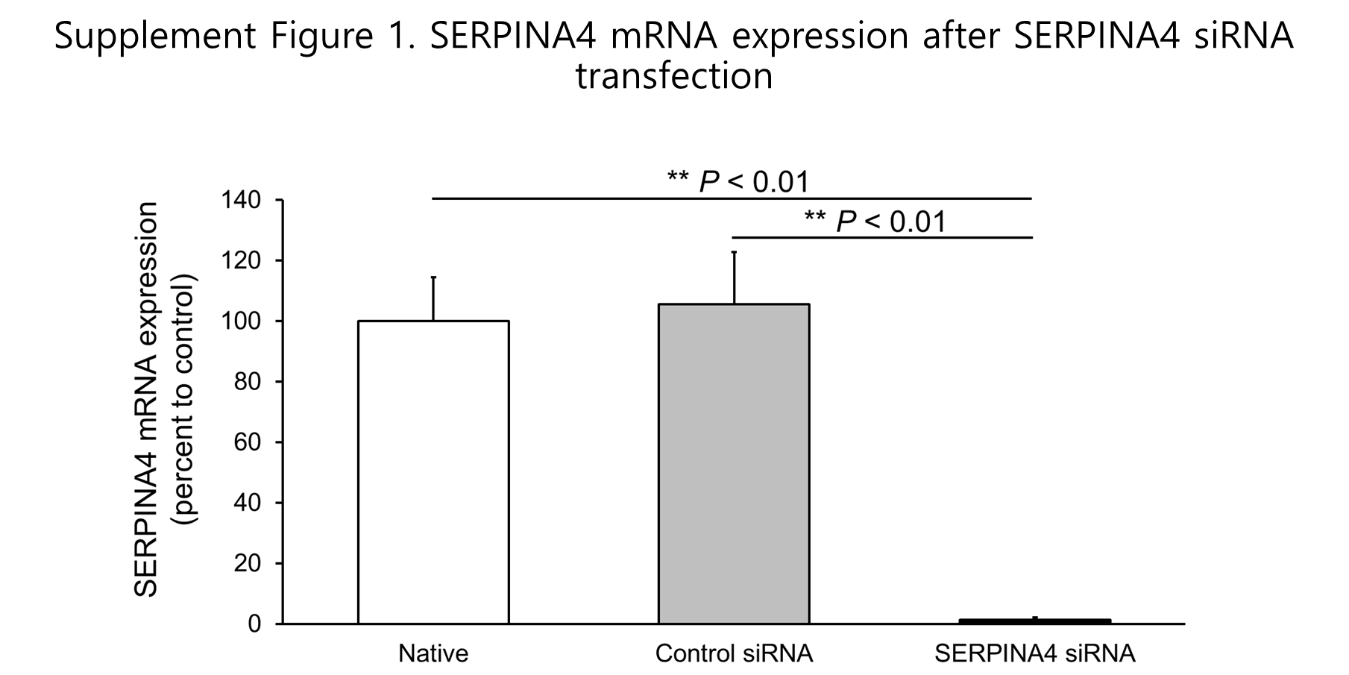


**Supplement 2.**

**A. Ischemic-reperfusion injury model and cell viability assessment**

To establish an ischemia-reperfusion injury model by oxygen-glucose deprivation (OGD) and reoxygenation (Reoxy), human neuronal cells were seeded in 24-well plates coated with poly D-L lysine, 40,000 cells were seeded per well and the cells were cultured for 48 hours. Then, Dulbecco's modified Eagle medium (DMEM), without glucose (11966025; Thermo Fisher Scientific, Waltham, MA), which is a glucose-deficient medium, was added to the culture plates, and the plates were incubated in a hypoxic chamber consisting of 95% nitrogen and 5% carbon dioxide (INCO108, Memmert, Schwabach, Germany) for 60 minutes. After oxygen-glucose deprivation treatment, the culture media without glucose was replaced with growth media, and reoxygenated for 23 hours in a chamber containing 95% air and 5% carbon dioxide (OGD/Reoxy). The OGD/Reoxy model mimics cerebral ischemia-reperfusion injury and is known to cause brain damage more rapidly than blocking the oxygen supply alone.^30^ Cell viability was measured using the tetrazole assay method (MTT assay) using modified 3-(4,5-dimethylthiazol-2-yl)-2,5-diphenyltetrazolium bromide. The cell viability was measured with/without OGD/Reoxy processes in control siRNA and kallistatin knockdown (SERPINA4 siRNA-transfected) human neuronal cells.

**B. Measurement of cell viability according to oxygen-glucose deprivation time**

First, to determine the appropriate OGD/Reoxy time in HCN-2 human neuronal cells, a glucose-deficient medium was added to HCN-2 cells, and the cells were incubated in a hypoxic chamber consisting of 95% nitrogen and 5% carbon dioxide for 30 minutes, 60 minutes and 90 minutes. After exposure, the medium was replaced with complete medium, and the cells were reoxygenated in a chamber containing of 95% air and 5% carbon dioxide for 23.5 hours, 23 hours, and 22.5 hours. Then, the cell viability was analyzed. The cell viability gradually decreased as the oxygen-glucose deprivation time increased from 30 minutes (p <0.05), 60 minutes (p <0.01), and 90 minutes (p <0.001) compared to the cell viability in the control group not treated with OGD/Reoxy.

Based on the results described above, there was a clear difference between the OGD/Reoxy groups and control group, and the appropriate oxygen-glucose deprivation time in HCN-2 cells was determined to be 60 minutes, considering the cell viability when the OGD/Reoxy experiment was performed in the kallistatin knockdown cells for longer time.

**
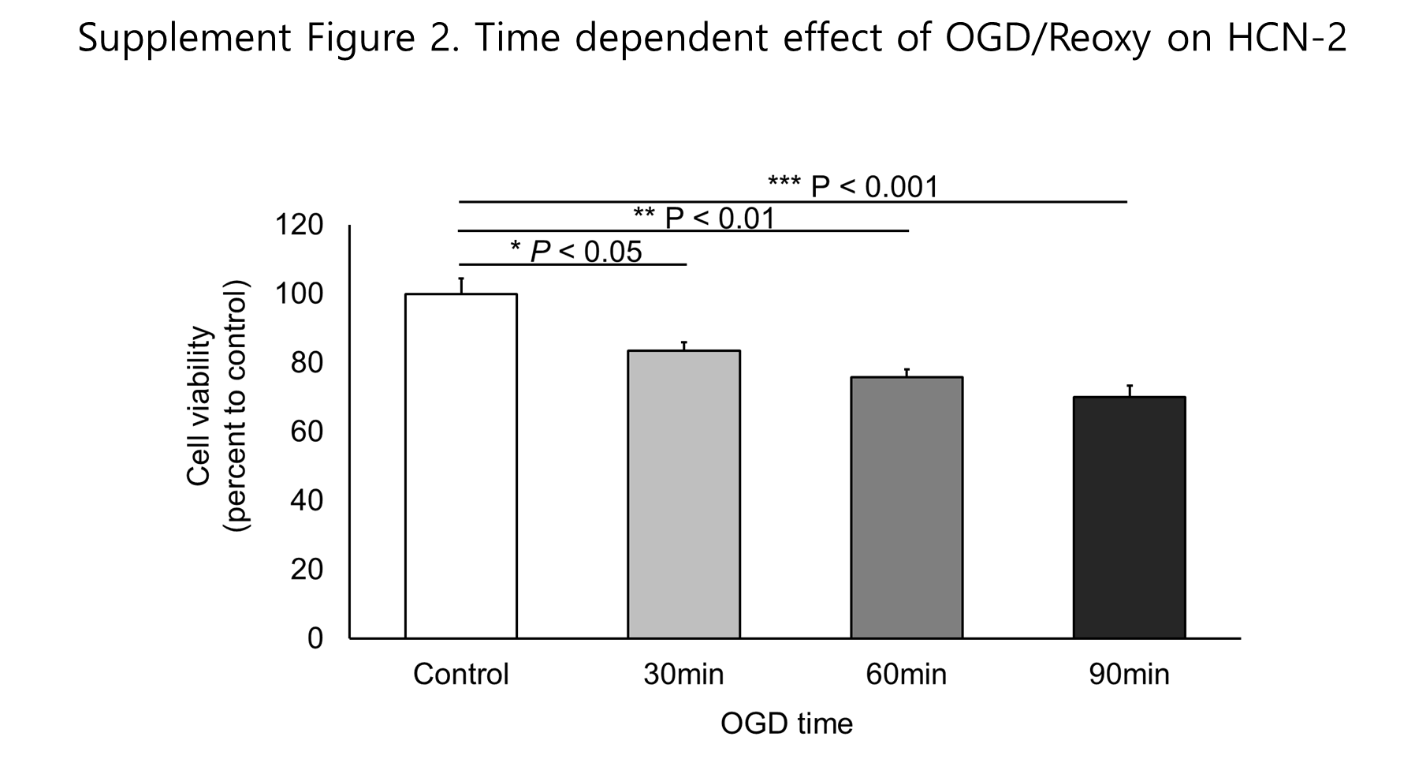
**

**Supplement 3.**

**A. Nox-1, Cleaved caspase 3 western blot results in control and kallistatin knockdown HCN-2 cells after exposure to OGD/Reoxy**

In order to measure intracellular oxidative stress, the expression of NADPH oxidase (Nox-1) was confirmed, and it was measured using western blot using anti-NOX1 antibody (Abcam, Catalog number: ab55831). Cleaved caspase 3 expression was confirmed to check apoptosis, and apoptosis was measured using anticaspase 3 (1:1,000; 9664; Cell Signaling, Danvers, MA) western blot. Through Western blot, it was confirmed that NADPH oxidase and cleaved caspase-3 were most expressed when the OGD/Reoxy process was performed in the knockdown neuronal cell group where kallistatin expression was suppressed. Original blots/gels are presented in Supplementary Figure 3.

**Supplement Figure 3. Nox-1, Cleaved caspase 3 western blot results in control and kallistatin knockdown HCN-2 cells after exposure to OGD/Reoxy.**

A. Nox-1 expression, B. Cleaved caspase 3 expression, C. β actin

**
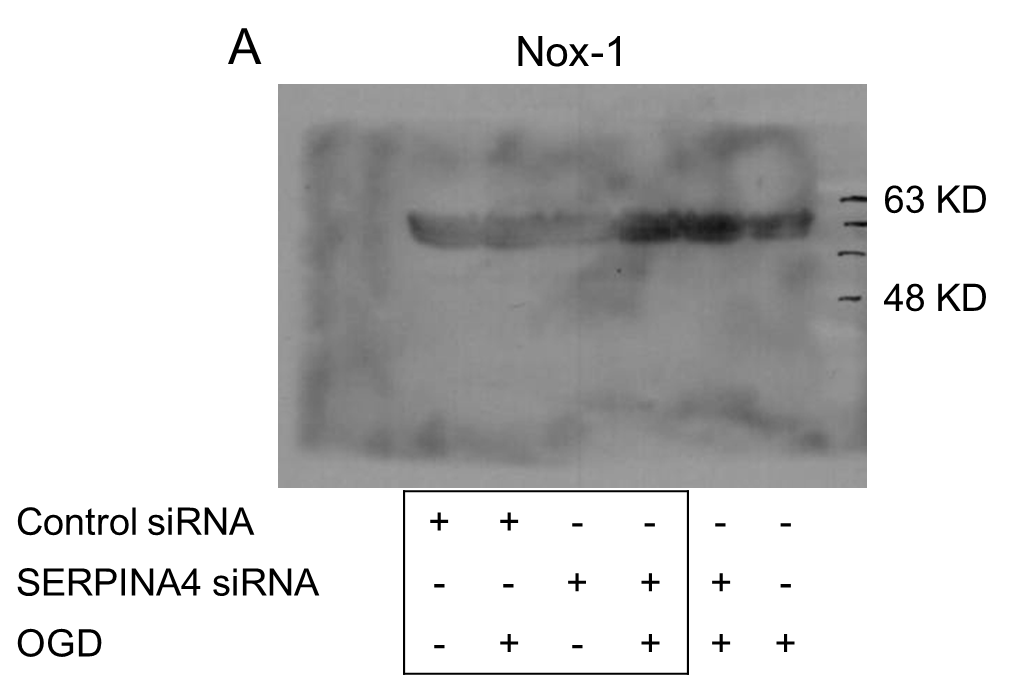
**

**
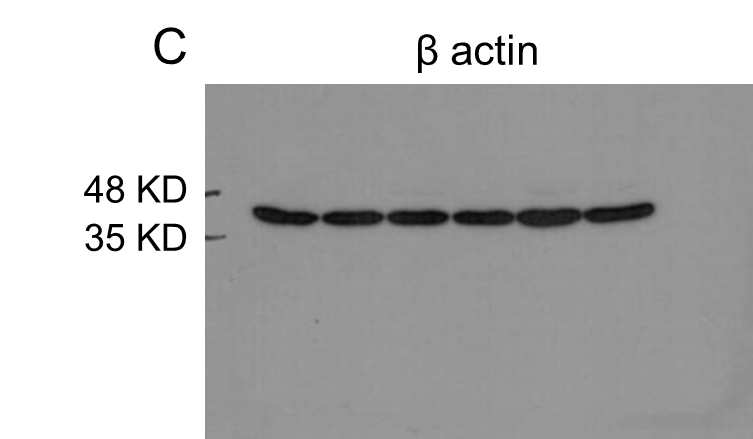

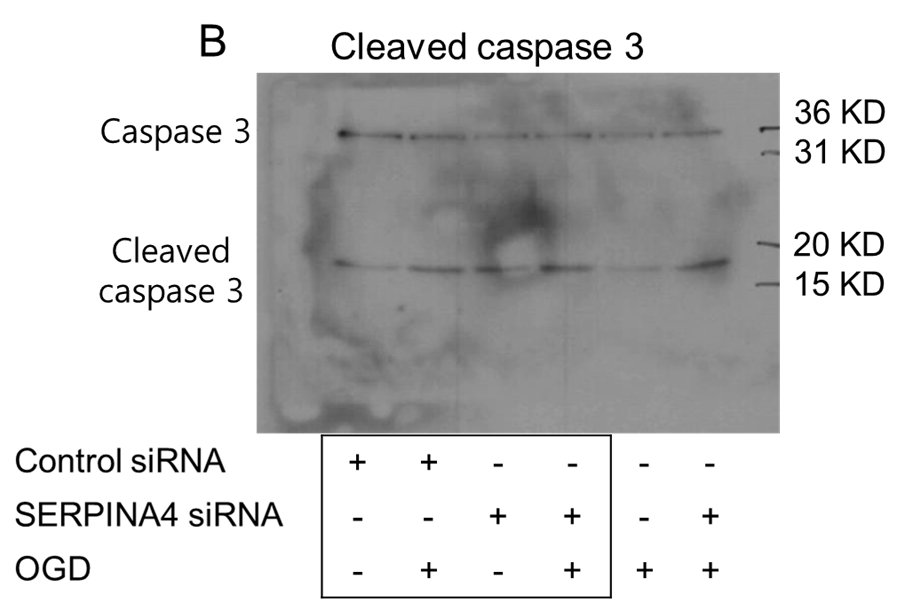
**
